# A case report and literature analysis of a rare nonossifying fibroma of the mandible

**DOI:** 10.1097/MD.0000000000044185

**Published:** 2025-09-05

**Authors:** Xihai Gao, Deshui Ran, Bao Zhong, Jingchao Han

**Affiliations:** a Binzhou Polytechnic, Binzhou, Shandong Province, China; b Department of Radiology, Jinan Second Hospital, Jinan, Shandong Province, China; c Department of Pathology, Experimental Diagnosis Department, Jinan Jinyu Medical Laboratory Center Co., Ltd., Jinan, Shandong Province, China; d Department of Medical Imaging, Central Laboratory of Jinan Stomatological Hospital, Jinan Key Laboratory of Oral Tissue Regeneration, Jinan, Shandong Province, China.

**Keywords:** cone beam CT, mandible, nonossifying fibroma, pathology, surgical treatment

## Abstract

**Abstract Rationale:**

Nonossifying fibroma (NOF) is one of the benign bone tumors in adolescents, and it rarely occurs in the jawbone. According to the site of onset, it is divided into the cortical type and the medullary type. Currently, there is no case report of medullary NOF in the mandible of the elderly. Through the description of the diagnosis and treatment experience of this case, combined with literature analysis, it provides help for clinical diagnosis and treatment.

**Patient concerns:**

The patient was a 69-year-old male who was treated for discomfort due to distension in the right mandibular first molar area. Clinical data collected in the past 10 years showed that the lesion grew slowly, and the imaging findings showed that the alveolar bone in the right mandibular first molar area showed uniform low-density imaging, the maxillary bone plate was locally eroded and damaged, and the lingual bone plate was significantly thinned.

**Diagnoses:**

Pathological sections showed that the lesions were mainly composed of a large amount of fibrous tissue, and the proliferated benign fibroblasts were arranged in a matted or swirling structure, in line with NOF manifestations.

**Interventions:**

The tumor was completely scraped off by surgical methods, and pathological examination was conducted.

**Outcomes:**

No discomfort was found at the surgical site during postoperative follow-up.

**Lessons:**

The incidence of NOF in the mandible of the elderly is extremely rare. It has a long growth cycle and no obvious clinical symptoms. Clinical diagnosis requires a full combination of imaging and pathology. Especially for the medullary type of NOF, the imaging manifestation is not characterized by cortical bone defect, but rather shows a tumor-like manifestation with uniformly low density similar to fibrous tissue. It has a certain invasiveness to the bone plate of the mandible. Surgical treatment should be carried out when necessary, and regular postoperative follow-up should be conducted.

## 1. Introduction

Nonossifying fibroma (NOF) is a benign tumor originating from the connective tissue of mature bone marrow, accounting for about 2% of primary bone tumors. It is more common in adolescent males aged 10 to 20 years. The metaphysis or diaphysis of long bones is the predilection site, among which the lower end of the femur and the upper end of the tibia have the highest incidence, and it is often a single-lesion disease.^[1,2]^ Some scholars have reported^[3,4]^ that traumatic stimulation, calcification due to malnutrition, and growth disorders may be the factors leading to NOF. Currently, reports of NOF occurring in the jawbone are still very rare. By searching the literature in the past 20 years, only 11 cases of patients have been reported, with the youngest being 6 years old and the oldest being 27 years old. The onset of this disease is slow, and there are generally no obvious clinical symptoms in the early stage. Larger lesions can lead to local soreness and swelling.

According to the site of onset, it is divided into the cortical type and the medullary type, with the latter being less common. The cortical type is prone to occur within or beneath the cortical bone of the metaphysis of long bones, growing eccentrically with a clear boundary; the medullary type is prone to occur in slender tubular bones and irregular bones, growing centrally, and both types can involve the diaphysis.^[2,5]^

Currently, there is no relevant literature report on cases of elderly patients with NOF occurring in the mandible. The initial imaging data of the case reported in this study confirmed that the onset time was approximately in 2014, and the clinical symptom of soreness occurred in 2024, with a disease course of up to 1 years. The postoperative pathological diagnosis was NOF. This study collected the relevant case data of the patient and analyzed it in combination with the relevant literature. The specific situation is reported as follows.

## 2. Case data

The patient, a 69-year-old male, presented with soreness and discomfort in the area of the right mandibular first molar and was admitted to Jinan Stomatological Hospital. The periapical radiograph showed a low-density lesion in the periapical area of the right mandibular first molar, and he was admitted to the hospital for treatment with a diagnosis of “mandibular mass.” The patient was in good health with no history of surgery and no family genetic disease.

### 2.1. Imaging follow-up findings

**Figure 1.**
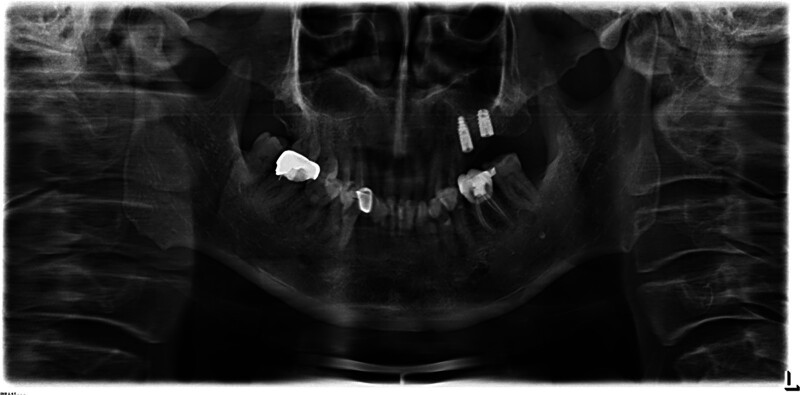
The oral panoramic radiograph shows the postoperative appearance of implant placement after the loss of the first and second maxillary molars on the left side, and a slight decrease in the density of the alveolar bone around the root apex of the distal root of the right mandibular first molar.

December 2014: An oral panoramic radiograph was taken in another hospital for dental implantation, which showed a slight decrease in the density of the alveolar bone in the distal periapical area of the right mandibular first molar, and no cystic-like lesion had formed yet (Fig. 1).

**Figure 2.**
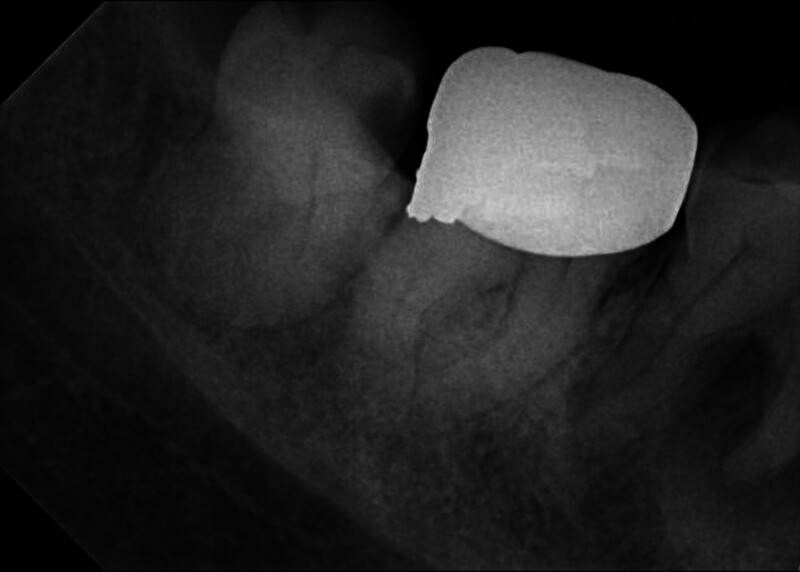
Digital periapical radiograph: the mesial part of the crown of the right mandibular third molar is defected and close to the pulp cavity, the crown of the second molar shows the appearance of a metal crown, and the periapical area of the first molar shows a patchy low-density shadow with an unclear boundary (the low-density shadow is larger compared with the oral panoramic radiograph in December 2014).

February 2017: A periapical radiograph was taken due to the caries of the right mandibular third molar, which indirectly showed a low-density shadow in the periapical area of the right mandibular first molar. The density was uneven and the boundary was unclear. The patient reported no discomfort in the area of the first molar (Fig. 2).

**Figure 3.**
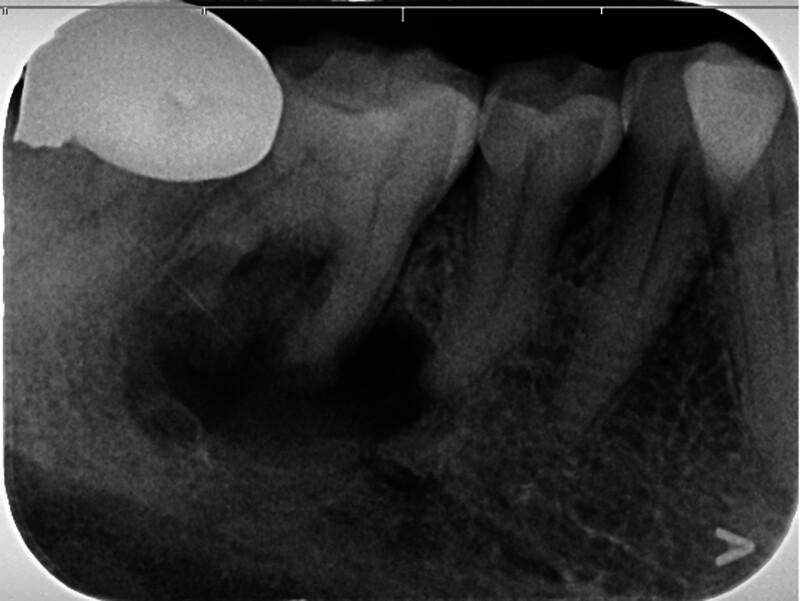
Digital periapical radiograph: a cyst-like low-density shadow is shown in the periapical areas of the right mandibular first molar and the second premolar. The density is uneven and the boundary is clear. There is a slight external root resorption at the root apex of the first molar (the lesion is significantly larger compared with that in February 2017).

June 2024: Due to the discomfort in the area of the right mandibular first molar, a periapical radiograph was taken, which showed a cyst-like low-density shadow in the periapical areas of the right mandibular first molar and the second premolar. The density was uneven and the boundary was clear. The lesion was significantly larger compared with that in February 2017 (Fig. 3).

**Figure 4.**
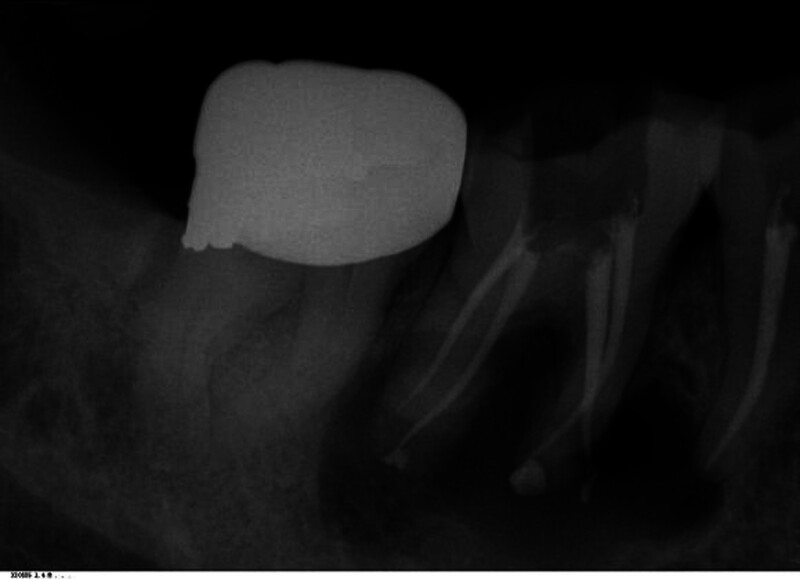
Digital periapical radiograph: a cyst-like low-density shadow is shown in the periapical areas of the right mandibular first molar and the second premolar, and a high-density shadow is shown filling the root canals.

June 2024: In order to preserve the right mandibular second premolar and the first molar during the operation, root canal treatment was performed on the right mandibular second premolar and the first molar before the operation, indicating good root canal filling (Fig. 4).

**Figure 5.**
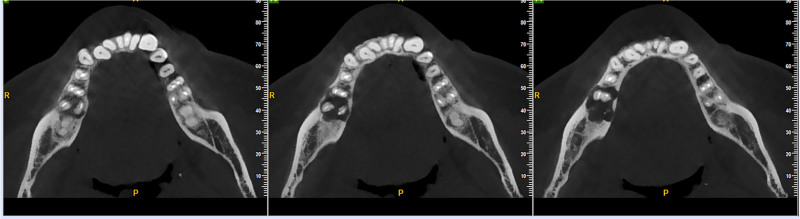
CBCT horizontal view: a patchy irregular low-density lesion in the periapical areas of the right mandibular second premolar and the first molar. The density inside the cyst is uniform, the boundary is clear, and it has a shallow lobulated appearance. The buccal bone plate is thinned due to expansive compression, and there is a small cyst-like erosion and destruction in the local lingual bone plate, with a slight external root resorption. CBCT = cone-beam computed tomography.

**Figure 6.**
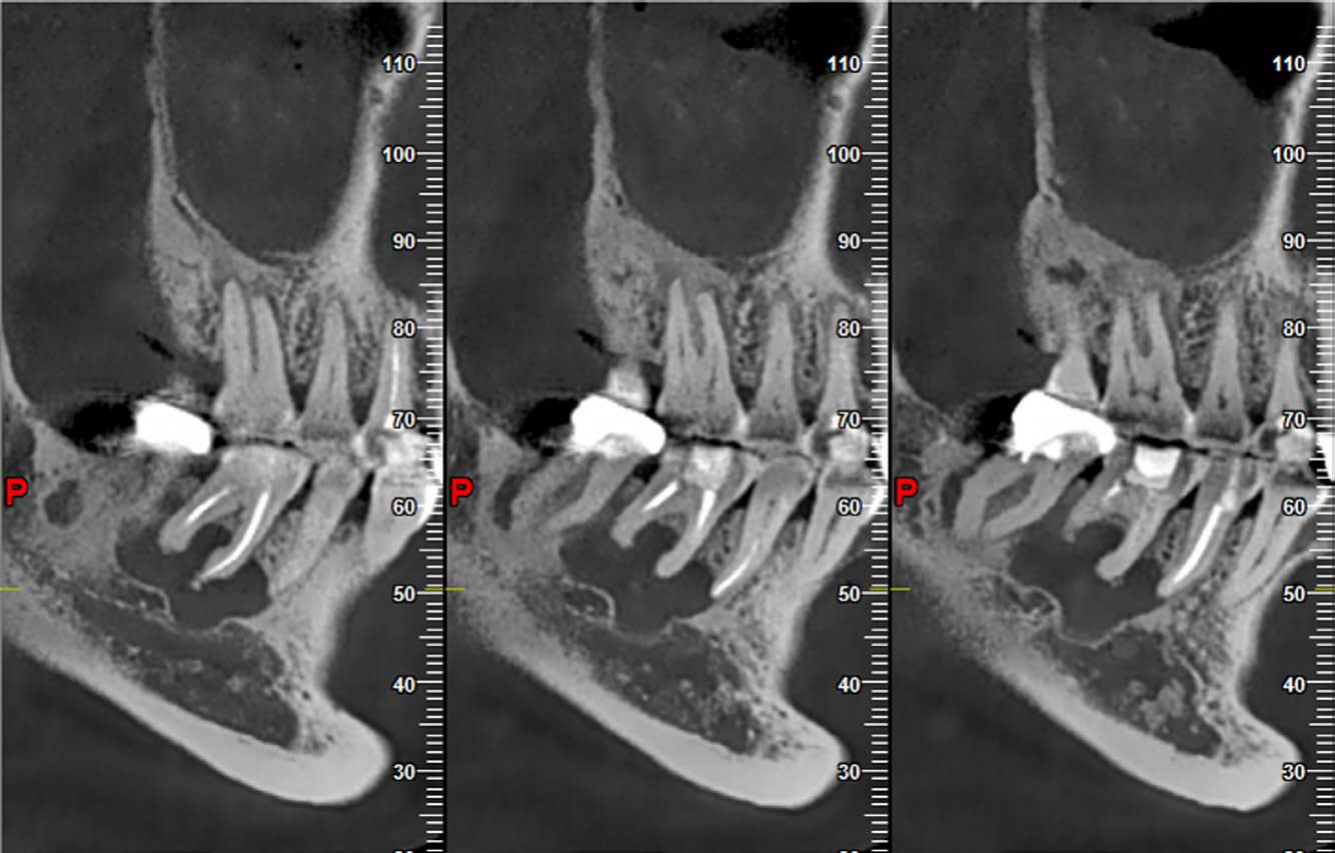
CBCT mesiodistal view: a thin sclerotic margin around the lesion, the shape is irregular with a shallow lobulated appearance, and it is adjacent to the mandibular canal below.

Cone beam computed tomography (CBCT) examination: in July 2024, a CBCT examination was performed before the operation, which showed a patchy irregular low-density lesion in the periapical areas of the right mandibular second premolar and the first molar. The maximum diameter was 19mm × 10mm × 10mm. The boundary was clear, and a thin sclerotic margin could be seen. The shape was irregular with a shallow lobulated appearance. The buccal bone plate was thinned due to expansive compression, and there was a small cyst-like erosion and destruction in the local lingual bone plate, with a slight external root resorption (Figs. 5 and 6).

### 2.2. Surgical methods

1. After anesthesia, multifunctional electric drill was used to make a horizontal bone incision from the right mandibular second premolar tooth to the right mandibular first molar tooth in the center below the root tip, forming a rectangular bone window to reveal the tumor; the tumor was completely scraped off and sent for pathological examination.
2. The bone wound edge was trimmed, the wound cavity was rinsed with a large amount of normal saline, filled it with medical collagen sponge, and then the incision was sutured.

### 2.3. Pathological examination

**Figure 7.**
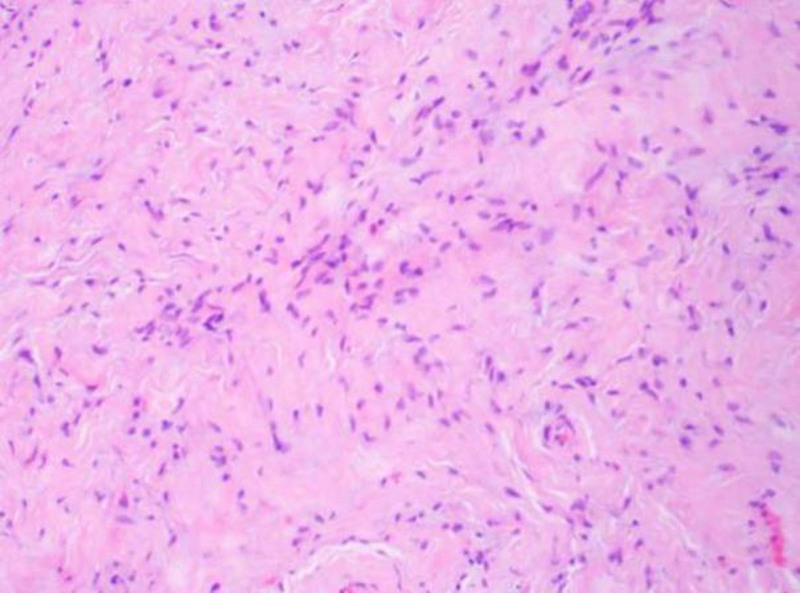
Pathological sections: under the microscope, there is a large amount of fibrous tissue, and the proliferated benign fibroblasts are arranged in a storiform or whorled structure (hematoxylin and eosin, ×100).

**Figure 8.**
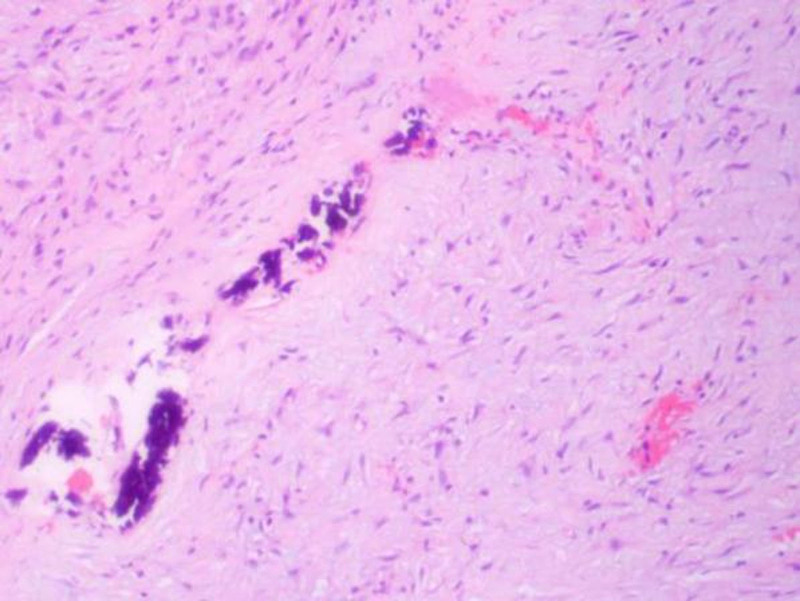
Pathological sections: the morphology of the hyperplastic benign fibroblasts is basically the same, mitosis is rare, and small foci of calcification can be seen (hematoxylin and eosin, ×100).

**Figure 9.**
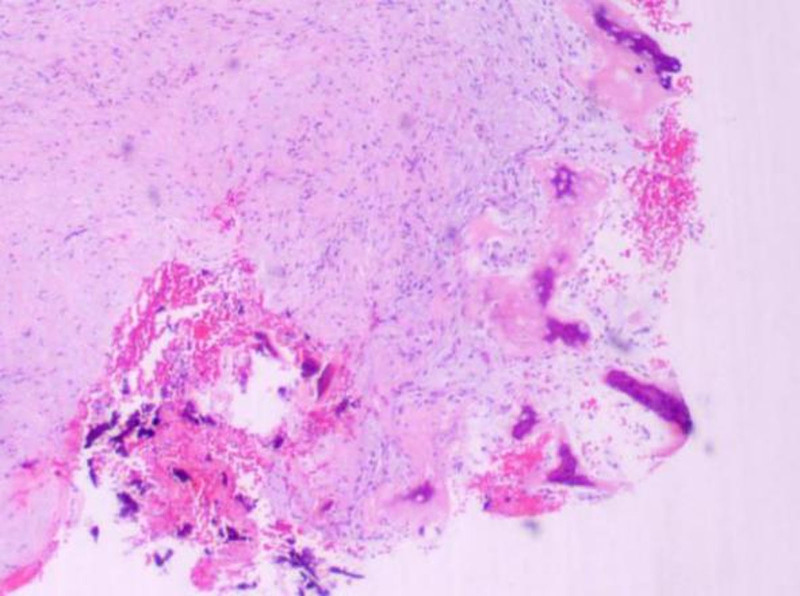
Pathological sections: a small amount of reactive hyperplasia of bone tissue is seen at the edge of the tissue, accompanied by bleeding, and osteoclast giant cells can be observed (hematoxylin and eosin, ×100).

**Figure 10.**
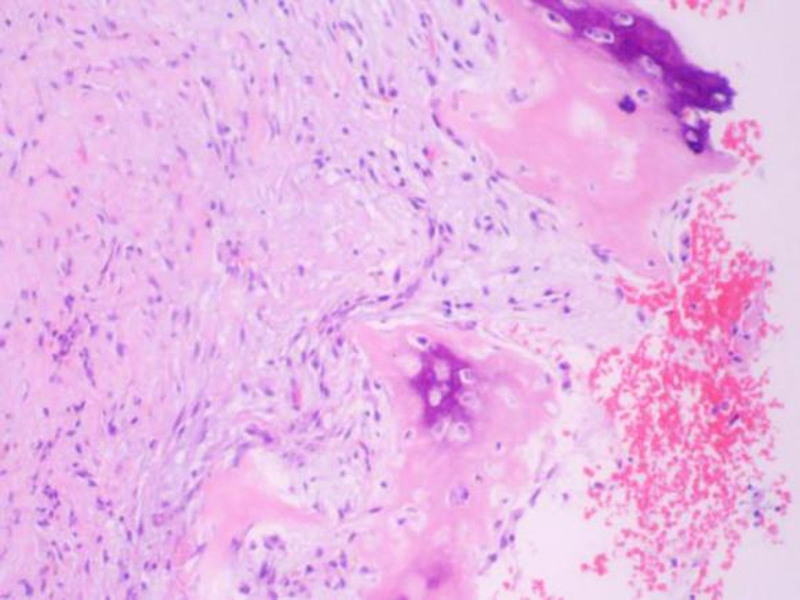
Pathological sections: a small amount of immature bone tissue and calcification can be seen around (hematoxylin and eosin, ×100).

**Figure 11.**
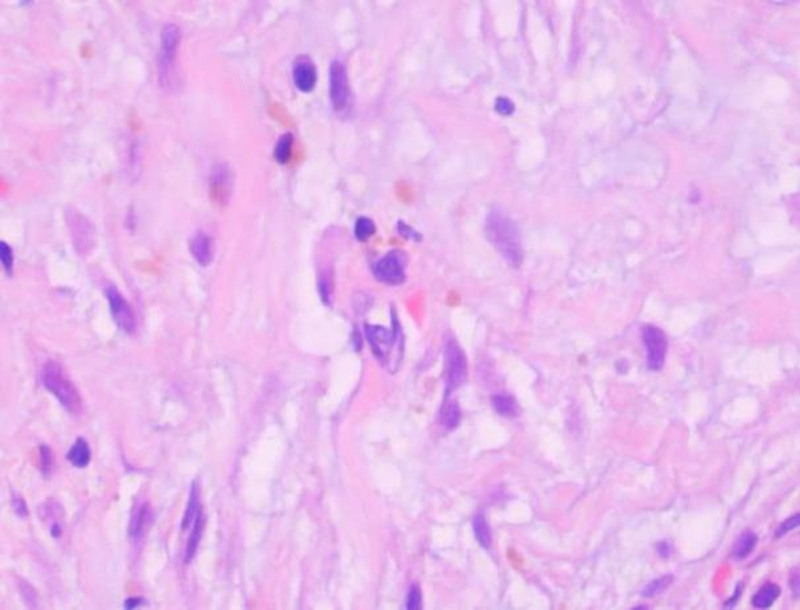
Pathological sections: the lesion contains foamy histiocytes, benign multinucleated giant cells and hemosiderin (HE × 400).

**Figure 12.**
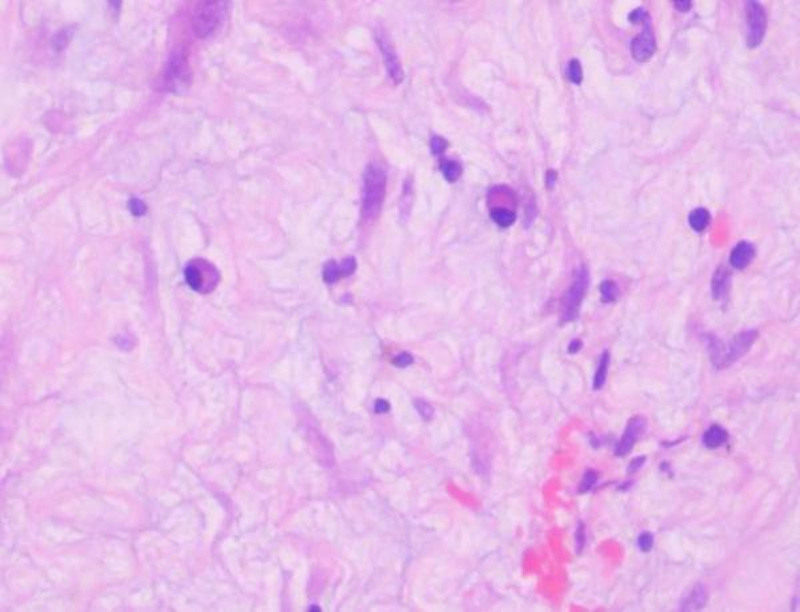
Pathological sections: Inflammatory cells and plasma cell infiltration can be seen in the focal area (HE × 400).

The resected mandibular lesion tissue was grayish-red and grayish-yellow. Multiple sections were made from the lesion. The pathological sections showed that the lesion was mainly composed of a large amount of fibrous tissue. The proliferating benign fibroblasts were arranged in a storiform or whorled pattern (Figs. 7 and 8). Foamlike histiocytes, benign multinucleated giant cells, and hemosiderin were present in the lesion (Fig. 9). Focal infiltration of inflammatory cells and plasma cells was observed (Fig. 10). A small amount of immature bone tissue and calcification were seen around the lesion (Figs. 11 and 12) in line with NOF manifestations.

### 2.4. Reexamination in January 2025

**Figure 13.**
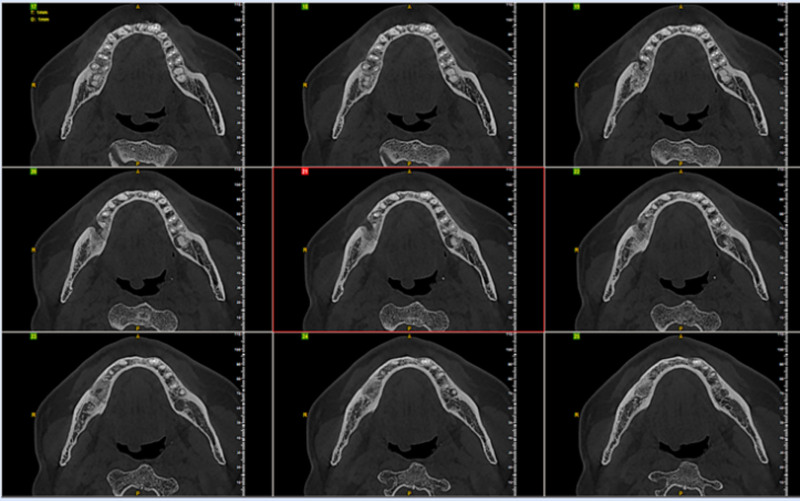
CBCT horizontal position: at the right mandibular surgical site, fresh bone tissue can be seen, with good shape. CBCT = cone-beam computed tomography.

The bone defect in the operative area of the right mandible has decreased. Fresh bone tissue has filled the defect, the trabecular bone structure is clear, and there is no sign of recurrence (Fig. 13). The patient was satisfied with the treatment and there was no discomfort in the surgical area.

## 3. Discussion

### 3.1. Imaging analysis of NOF

According to the location of NOF, X-ray and CT images classify it into the cortical type and the medullary type. The cortical type, also known as the eccentric type, mostly occurs in the cortical bone on one side, growing eccentrically. It presents as a unilocular or multilocular expansile low-density translucent area with a clear boundary. The lesion is consistent with the long axis of the bone and can protrude into the medullary cavity. There is often a “scallop-like” sclerotic zone on the medullary cavity side. The medullary type, also known as the central type, is more common in the metaphysis of long tubular bones, showing central expansion and eroding the cancellous bone. It is manifested as a unilocular or multilocular translucent area with uneven density. In the case of a multilocular lesion, there may be residual sclerotic bone ridge bands inside the cysts, and the cortex can become thinner and expand.^[5–7]^ However, since the mandible is an uncommon location and lacks specific manifestations, especially it is very difficult to differentiate the medullary NOF of the jawbone from desmoplastic fibroma, odontogenic fibroma, ameloblastic fibroma, etc. It is usually impossible to make a diagnosis solely based on imaging before surgery, and a pathological diagnosis is required in combination.

In this case, the lesion is located in the periapical area of the right mandibular first molar, growing in a central type. Most of the edges of the lesion show a thin sclerotic zone, which is consistent with the manifestation of medullary NOF. However, it should be noted that there are small cystic erosions on the lingual bone plate of this lesion, involving both the buccal and lingual bone plates. It is not that the thinning of the lingual bone plate is solely caused by expansive external pressure. The local thinning of the cortical bone, from the perspective of imaging analysis, indicates that the medullary NOF has a certain degree of invasiveness. Mannan et al reported a case of a 15-year-old boy with an expanding osteolytic lesion in the left mandible.^[8]^ Tabrizi et al reported a case of NOF secondary to an aneurysmal bone cyst of the mandibular condyle, which showed osteolytic destruction and an unclear boundary with the surrounding soft tissues.^[9]^ Tabassum et al reported a case of a 15-year-old girl, and the CBCT showed bilateral multilocular low-density lesions, with expansion being the main manifestation of the jawbone.^[4]^ The imaging of the 16-year-old female NOF case reported by Pandiar et al suggested a certain degree of invasiveness, and it was misdiagnosed as a unicystic ameloblastoma or an odontogenic keratocyst before surgery.^[6]^ Through the author’s research and the cases reported in the literature, it can be found that the destruction of the bone by NOF in the jawbone is not limited to expansion, and it can also lead to invasive manifestations or osteolytic destruction.

### 3.2. Pathological analysis of NOF

Referring to the diagnostic criteria of clinical pathology of bones and joints,^[10]^ under the microscopic observation of a typical NOF, it can be found that the tumor is composed of a very abundant number of spindle-shaped fibroblasts, arranged in layers or whorls. Hemosiderin granules or lipid-like precipitates can also be found in the giant cells and fibroblasts; foam cells or xanthoma cells may also be formed. There are varying amounts of collagen fibers between the cells. There is no osteogenic activity in the tumor, and reactive bone hyperplasia can occur in the bone tissue adjacent to the tumor. Bailey et al reported^[11]^ that NOF has the following 5 pathological characteristics: a wheel-like, layered, fibrous connective tissue matrix; spindle-shaped fibroblasts; multinucleated giant cells; the presence of foam cells or xanthoma cells, which are considered to be transformed from fibroblasts; the lack of bone formation at the lesion site. The pathology of this case is consistent with the typical manifestations of NOF and also has the 5 pathological characteristics of NOF reported by Bailey et al. In terms of imaging, this lesion is closely adjacent to the mandibular first molar, and it needs to be differentiated from odontogenic cystic lesions or tumors. However, after multilayer pathological section examination, no components such as odontogenic epithelial tissues and enamel that support odontogenic cystic lesions or tumors were found. In addition, it is very difficult to differentiate this disease from desmoplastic fibroma of the jawbone, and it is necessary to closely combine it with imaging and clinical manifestations.

### 3.3. Differential diagnosis

1. Odontogenic fibroma is a benign tumor characterized by the presence of varying amounts of active odontogenic epithelium in the mature fibrous stroma.^[12]^ X-ray and CBCT mainly show low-density projections, occasionally with punctate high-density calcifications, and some may contain teeth. Under the pathological microscope, the presence of inactive odontogenic epithelial rest cords or irregular epithelial islands in the mature fibrous tissue is the key to the differential diagnosis from NOF.^[12,13]^
2. Ameloblastic fibroma is a rare benign tumor that does not contain dental hard tissues and is composed of odontogenic epithelium and odontogenic mesenchyme similar to the dental papilla.^[14]^ The imaging manifestations are both low-density transmission shadows, which cannot be differentiated from those of NOF. However, under the pathological microscopic observation, it can be found that the neoplastic epithelium is arranged in cords or masses, and the mesenchymal component is composed of immature connective tissue similar to dental papilla cells,^[13,15,16]^ which can be used as the key to the differential diagnosis from NOF.
3. Desmoplastic fibroma of bone (DMPF) is composed of mildly atypical spindle cells and a large amount of collagen produced by them.^[12]^ In 1958, Jaffe first separated it from desmoid fibroma of soft tissues and other central fibrous lesions and named it DMPF.^[12]^ The latest classification of bone tumors by World Health Organization (2013) has classified DMPF from benign tumors into intermediate fibroblastic tumors of bone. Its X-ray manifestations are unilocular or multilocular transmissive lesions with clear or blurred boundaries, some of which are map-like, and there is a narrow transition zone. A sclerotic margin is rarely seen. Pseudotrabeculae can be seen in the lesion, and the presence of “root-like” bone ridges in the lesion area is an important feature of DMPF,^[13,17,18]^ while NOF generally has a sclerotic margin, and there are no “root-like” bone ridges and pseudotrabecular structures. In terms of pathology, DMPF has a single cell component, mainly composed of fibroblasts and dense collagen fibers, and the tumor tissue grows invasively,^[19]^ while NOF contains varying amounts of xanthoma cells, macrophages, and multinucleated giant cells, and the tumor tissue grows expansively.

### 3.4. Treatment of NOF

Some clinical reports suggest that NOF and cortical bone defects are the same type of disease. NOF has a certain degree of self-healing ability. It can be dynamically observed without intervention when there are no clinical symptoms.^[20,21]^ However, when obvious pain, swelling, and other clinical symptoms appear, and there is a tendency to develop pathological fractures and malignant transformation, surgical treatment is required.^[22,23]^

According to the previous clinical data in this case, clinically, it is judged that the low-density lesion of the right mandible has a growing trend and invades the mandibular bone plate, which has the clinical manifestations of a jaw tumor and meets the indications for surgical treatment. In the case of a 15-year-old boy with mandibular NOF reported by Mannan et al, the lesion was curetted, and a microplate was implanted at the resection site due to the large scope of the lesion. After 2 years of continuous follow-up, there was no recurrence.^[8]^ In the case of a 15-year-old female reported by Tabassum et al, the lesion was successfully treated by surgical curettage, but recurrence of the right lesion was found during the 2-year follow-up after surgery, and a second surgical intervention was carried out.^[4]^ It indicates that it is very necessary to adopt surgical treatment and regular postoperative follow-up for NOF of the jawbone according to clinical symptoms and imaging manifestations.

### 3.5. Conclusion

The incidence of NOF in the jawbone is extremely rare, especially in elderly patients. This case fills the gap in the report of NOF in the jawbone of the elderly. Clinical diagnosis requires a full combination of imaging and pathology. Through the research and analysis of this case, for NOF in the mandible of the elderly, especially the medullary type of NOF, the imaging manifestation is not characterized by cortical bone defect, but rather shows a tumor-like manifestation with uniformly low density similar to fibrous tissue. Although NOF grows relatively slowly, it will invade the bone plate as the lesion grows. Whether it is a child or an elderly patient, surgical treatment should be carried out when necessary according to the risk of the lesion, and regular follow-up is required after surgery.

## Acknowledgments

The authors would like to thank Central Laboratory, Jinan Key Laboratory of Oral Tissue Regeneration, Jinan Stomatological Hospital, Jinan, China.

## Author contributions

**Conceptualization:** Deshui Ran.

**Resources:** Bao Zhong.

**Writing – original draft:** Xihai Gao.

**Writing – review & editing:** Jingchao Han.
